# Resin from *Virola oleifera* Protects Against Radiocontrast-Induced Nephropathy in Mice

**DOI:** 10.1371/journal.pone.0144329

**Published:** 2015-12-16

**Authors:** Igor Santos Fonte Bôa, Marcella Leite Porto, Ana Claudia Hertel Pereira, Jean Pierre Louzada Ramos, Rodrigo Scherer, Jairo Pinto Oliveira, Breno Valentim Nogueira, Silvana Santos Meyrelles, Elisardo Corral Vasquez, Denise Coutinho Endringer, Thiago Melo Costa Pereira

**Affiliations:** 1 Pharmaceutical Sciences Graduate Program, Vila Velha University (UVV), Vila Velha, ES, Brazil; 2 Laboratory of Translational Physiology, Health Sciences Center, Federal University of Espirito Santo, Vitoria, Brazil; 3 Department of Morphology, Health Sciences Center, Federal University of Espirito Santo, Vitoria, Brazil; 4 Federal Institute of Education, Science and Technology (IFES), Vila Velha, ES, Brazil; Emory University, UNITED STATES

## Abstract

Contrast-induced nephropathy (CIN) is an iatrogenic medical event for which there is not yet a successful therapy. Increasing evidence in rodents has suggested that this disease is associated with renal tubular and vascular injury that is triggered directly by oxidative stress. In the present study, we evaluated whether the antioxidant resin from *Virola oleifera* (RV) could attenuate renal damage in an experimental mouse model of CIN. Adult male Swiss mice were divided into six groups and pre-treated orally with RV (10, 100 and 300 mg/kg), N-acetylcysteine (200 mg/kg) or vehicle for 5 days before the induction of CIN and Control group. Renal function was assessed by measuring plasma creatinine and urea levels. Additionally, renal oxidative stress and apoptosis/cell viability were determined with flow cytometry. Finally, kidney tissues were sectioned for histopathological examination. In this CIN model, pre-treatment with RV improved renal function, lowered the mortality rate, and reduced oxidative stress and apoptosis in both the medulla and cortex renal cells in a dose-dependent manner. Moreover, the RV treatment had beneficial effects on kidney histopathology that were superior to the standard treatment with N-acetylcysteine. These data suggest that because of its antioxidative and antiapoptotic effects and its ability to preserve renal function, resin from *Virola oleifera* may have potential as a new therapeutic approach for preventing CIN.

## Introduction

Contrast-induced nephropathy (CIN) is an iatrogenic event associated with increased morbidity and mortality, particularly in vulnerable clinical subpopulations (e.g., the elderly and patients with kidney, cardiac and/or diabetic disease) who have been subjected to diagnostic and interventional procedures [1,2]. Paradoxically, although the use of iodinated contrast media is essential in several diagnostic imaging techniques, strategies to prevent CIN are still inadequate [2–4], which exposes patients to the risks of long-term loss of kidney function and associated medical expenses [5]. Therefore, novel strategies for the prevention of CIN are fundamental in order to avoid kidney damage as well as minimize economic costs [3,6].

It is well-known that the causes of CIN are multifactorial, but the exact pathogenesis underlying this disease is not yet fully understood [2,6]. A combination of renal tubular and vascular injury, which is caused by the direct and indirect effects of reactive oxygen species (ROS) [3,7–9], is a critical determinant of the proximal tubular damage that has been observed histologically in both experimental [10,11] and clinical [1,2,12] CIN. N-acetylcysteine (NAC), ascorbic acid (vitamin C) and α-tocopherol (vitamin E) have been tested for their ability to prevent CIN because of their antioxidant properties [9,11,13]. However, inconsistent results have been found in several clinical trials, which has led to the search for other candidate renoprotective substances [1,9,14–16].

*Virola oleifera* (Schott) A. C. Smith, Myristicaceae, which is popularly known as “bicuíba”, “bocuva”, “bicuíva” or “candeia-do-caboclo”, is a tree found in the Atlantic forest [17]. When grated, the bark produces a resin that is commonly used for healing purposes [18]. This resin contains a mixture of several phenolic and flavonoid compounds, which exhibits strong antioxidant properties [19]. Therefore, we hypothesized that resin from *Virola oleifera* (RV) could attenuate the pathology that characterizes renal injury in an experimental model of CIN. The confirmation of this hypothesis may identify a novel approach for preventing CIN.

## Materials and Methods

### Resin material

The resin from *Virola oleifera* (Schott) A. C. Smith was collected in the district of Fazenda Guandu in the city of Afonso Claudio (Espirito Santo, Brazil, S20° 13490' W 041° 06692'). The plant material was verified by D.Sc. Luciana Dias Thomaz, Department of Botany, Federal University of Espirito Santo, where the voucher specimen was deposited (VIES 19648). The plant was collected with authorization and in accordance with the Brazilian Law (Resolution 29, 12/06/2007), which states that no special permission is necessary to collect samples of essential oil or fixed oil or when the tested material remains similar to the raw material (Provisional Statement 2.186–16, 08/23/2001). Because the material employed in the present study is resin, we are in accordance with the aforementioned law. Fluid exudate was obtained from 0.5 cm deep incisions in the tree trunk. The exudate was collected in aseptic plastic containers and transferred into an amber glass vial and kept at 4°C until the final analysis. The fluid exudate was dried at 40°C, grained, and the final yield obtained was 24 g of dried resin.

### Identification and quantification of phenolic acids by LC/MS

A liquid chromatograph coupled to a triple quadrupole (Q1q2Q3) mass spectrometer (Agilent 1200 HPLC equipped with API3200—Applied Biosystems) was used for LC/MS analysis. All separations were performed on an Agilent Eclipse C18 column (150 mm x 4.6 mm, 5 mm) at 35°C. The mobile phase consisted of (A) an aqueous solution of formic acid (0.05% v/v) and (B) acetonitrile (0.3 mL/min) using an elution gradient of 10–60% of B from 0–8 min, 60–90% of B from 8–12 min, 90–100% B from 12–15 min. The balance time was 6 min. The MS nebulizer pressure was 50 psi. The gas temperature was 650°C and the capillary voltage was 5500 V.

Identification of the chemical constituents present in the dried resin was performed using LC/MS/MS. Briefly, samples were diluted in HPLC grade methanol (Sigma Aldrich, St. Louis, MO, USA, 1 mg/mL) and filtered. The compounds were identified by comparing their retention times and spectra with those from standard solutions. Quantification was performed using external curves of seven points for the following standards: ferulic acid (linear range: 1.1–0.0172 μg/mL, LD: 0.00216 μg/mL; LQP: 0.0172 μg/mL), caffeic acid (linear range: 0.5–0.00781 μg/mL, LD: 0.0026 μg/mL; LQP: 0.0078 μg/mL), rosmarinic acid (linear range: 2.6–0.04063 μg/mL; LD: 0.0382 μg/mL; LQP: 0.0406 μg/mL), gallic acid (linear range: 9.2–0.14375 μg/mL; LD: 0.0030 μg/mL; LQP: 0.1438 μg/mL), apigenin (linear range: 0.2875–0.00449 μg/mL, LD: 0.0025 μg/mL; LQP: 0.0045 μg/mL) and quercetin (linear range: 0.03125–2 μg/mL; LD: 0.0117 μg/mL; LQP: 0.0313 μg/mL). The limits of detection and quantification were 3 and 5 times the noise signal, respectively.

### Determination of antioxidant activity

The antioxidant activity of the RV was determined according to Scherer and Godoy [20] by measuring free-radical scavenging activity using 2,2-diphenyl-1-picrylhydrazyl (DPPH•). The equation of the line used for the sample showed an r^2^ = 0.99. The antioxidant activity index (AAI) was calculated by dividing the final concentration of DPPH by the 50% inhibitory concentration (IC_50_). The results were expressed in AAI ± standard deviation and IC_50_ ± standard deviation. The AAIs for a standard (ferulic acid) and a synthetic antioxidant, butylhydroxytoluene (BHT), were determined as previously described [20]. The activity of each sample was classified as weak (AAI <0.5), moderate (0.5 <AAI <1.0), high (1.0 <AAI <2.0) and very high (AAI> 2, 0) according to the index developed by Scherer and Godoy [20].

### Animals

Adult male Swiss mice *Mus musculus* weighing 25 to 40 g were obtained from the Experimental Monitoring Laboratory of Vila Velha University (UVV), Espirito Santo, Brazil. The animals were fed a normal chow diet and water *ad libitum* and housed under standard conditions with a 12 h dark-light cycle and temperature at 22°C. All biological assays were performed according to the international principles from the National Institutes of Health Guide for the Care and Use of Laboratory Animals and the Brazilian Society of Experimental Biology. The study was previously approved by the Ethics, Bioethics and Animal Welfare Committee of Vila Velha University (EBAW-UVV 268/2013). Humane end points were set according to the OECD—Guidance Document on the Recognition, Assessment, and Use of Clinical Signs as Humane Endpoints for Experimental Animals Used in Safety Evaluation (https://www.aaalac.org/accreditation/RefResources/RR_HumaneEndpoints.pdf). In a pilot study, we observed that the survival rate for CIN was ~60%, and the critical post-intervention period was between 20 and 24 hours. Therefore, during this period the humane endpoints were determined by paying special attention to occurrences of decreased activity followed by a progression to no response to touch, a drop in body temperature, sunken eyes or a hunched posture. When all efforts to minimize the suffering of the animals failed, the animals were sacrificed using an overdose of sodium thiopental.

### Treatments and experimental protocol (Induction of CIN in mice)

Mice were randomized into six groups: group 1: control; group 2: contrast-induced nephropathy (CIN); group 3: N-acetylcysteine (NAC) at 200 mg/kg; group 4: RV 10 mg/kg (V10); group 5: RV 100 mg/kg (V100); group 6: RV 300 mg/kg (V300). These doses of resin were chosen based on previous studies (unpublished data). After 5 days of oral pretreatment with vehicle (groups 1 and 2), NAC (group 3) and RV (V10, V100 and V300), the CIN procedure was performed.

The innovative CIN procedure in mice was adapted from protocols previously validated by Billings et al. [10] and Lee et al. [21]. In brief, after overnight (16 h) water deprivation and inhibition of nitric oxide and prostaglandin synthesis, mice were injected intraperitoneally (i.p.) with a low-osmolar monomeric iodinated radiocontrast medium, ioversol (Optiray, 320 Mallinckrodt Medical, Inc., St. Louis, MO, 1.5 g iodine/kg). For inhibition of nitric oxide synthase and cyclooxygenase, mice were injected with L-NG-nitro arginine methyl ester (L-NAME, 10 mg/kg i.p., dissolved in 0.9% saline) and indomethacin (10 mg/kg i.p, dissolved in dimethylsulfoxide), respectively, 15 minutes before the ioversol injection. After the ioversol injection, the animals had free access to food and water. The animals were euthanized 24 h later using an overdose of sodium thiopental (Cristalia, Sao Paulo, Brazil, 200 mg/kg, i.p.) for biochemical, cytological and histopathological examinations.

### Blood and kidney samples

Venous blood was collected from the right ventricle for serum urea and creatinine measurements using an automatic spectrophotometric analyzer from a clinical analysis laboratory (Tommasi Laboratory, Vitoria, ES, Brazil). Immediately afterward, the tissues were perfused with cold phosphate-buffered saline (PBS, pH 7.4, 0.1 M) through the left ventricle, and both kidneys were excised. In the kidney samples designated for flow cytometry analysis, the cortex was separated from the medulla before the assay. The renal cells were prepared based on previous and standardized studies in our laboratory [22,23]. Briefly, the tissues were grossly minced using surgical scissors and were incubated with an isolation solution containing trypsin (Sigma-Aldrich) to dissociate the cells. Next, the cell suspension was filtered through a nylon screen (BD, Becton Dickinson, San Jose, CA, USA, Falcon 70 μm) to remove the cellular debris. Finally, the samples were washed twice in PBS and stored at -80°C until further analysis. For histopathological examination, the kidney samples were fixed in formaldehyde (10%), and histological slides were prepared and counterstained with hematoxylin-eosin (HE).

### Cell viability

Cell viability was evaluated using propidium iodide (PI) staining exclusion. A total of 10^6^ cells were incubated with 2 μL of PI for 5 min in the dark at room temperature. The cells were washed with PBS and analyzed with a FACS-Canto II flow cytometer (BD). For viability quantification, samples were acquired in triplicate, and 10,000 events were used for each measurement. Cells were excited with a wavelength of 488 nm, and PI fluorescence was detected using a 585/42 bandpass filter. The data are expressed as the percentage of unstained/viable cells [24].

### Measurement of ROS and hROS production using flow cytometry

The ROS analysis was performed using flow cytometry as previously described [25]. Dihydroethidium (DHE, 160 μM) and 2’,7’-dichlorofluorescein diacetate (DCF, 20 mM) were added to the cell suspension (10^6^ cells) and incubated at 37°C for 30 min, in the dark, to estimate intracellular •O_2_^-^ or H_2_O_2_ concentrations, respectively. Highly reactive oxygen species (hROS), such as hydroxyl radicals and peroxynitrite, were selectively detected by 2-[6-(4'-hydroxy)phenoxy-3H-xanthen-3-on-9-yl]benzoic acid (HPF) as designed and described by Setsukinai et al. [26]. Cells were then washed, resuspended in PBS and analyzed with flow cytometry (FACSCanto II). Data were acquired using FACSDiva software (BD) and overlay histograms were analyzed using FCS Express software trial (De Novo). For quantification of DHE, DCF and HPF fluorescence, samples were acquired in duplicate, and 10,000 events were used for each measurement. Cells were excited at 488 nm, DHE fluorescence was detected using a 585/42 bandpass filter and DCF/HPF fluorescence was detected using a 530/30 bandpass filter. The data are expressed as median fluorescence intensity (MFI, in a.u.).

### Apoptosis

Apoptotic kidney cells were quantified by annexin V-FITC and PI double staining using an annexin V-FITC apoptosis detection kit (BD). In brief, cortex and medulla cells were washed twice with PBS and adjusted to 500 μL of the binding buffer (5x10^5^ cells). Then, the cell suspensions were incubated with annexin V–FITC and PI for 15 min at room temperature (25°C) in the dark. Finally, cells were analyzed with a FACSCanto II (BD) flow cytometer. Viable cells with positive staining for annexin V (Q2+Q4) were considered apoptotic cells.

### Kidney histology

The renal tissues were embedded in paraffin, and sections (4 μm) were stained with HE. Tubulointerstitial injury (TILI), which is characterized by tubular atrophy, thickening of the basement membrane, dilatation and protein cast, was assessed by semi-quantitative analysis in accordance with Wu et al. [27] and Liu et al. [28]. Thirty cortical and medullary fields from each animal were examined at 400x magnification and graded according to a scale from 0 to 4: Grade 0, no tubulointerstitial injury; Grade 1, <25% of the tubulointerstitium injured; Grade 2, 25–50% of the tubulointerstitium injured; Grade 3, 51–75% of the tubulointerstitium injured; and Grade 4, 76–100% of the tubulointerstitium injured. All sections were examined by researchers blind to the experimental groups of the samples.

### Scanning electron microscopy (SEM)

Kidney samples from different groups were fixed in paraformaldehyde (2%)-glutaraldehyde (2,5%) cacodylate buffer solution (0.1 M; pH 7.2) for 24 h, washed with cacodylate buffer, postfixed in a solution of 1.25% potassium ferrocyanide for 1 h at room temperature and washed again in cacodylate (0.1 M) buffer and ultrapure water. Longitudinal sections from kidney samples were infiltrated with glycerol solutions (rising from 15% to 30% over 5 minute intervals) to prevent the formation of ice crystals in the sample. After 3 h in glycerol (30%)-cacodylate (0.1 M) buffer solution, the samples were frozen to -80°C and fractured using cooled tweezers. Fractured samples of kidney were washed in cacodylate buffer and ultrapure water and dehydrated in ascending grades of ethanol, subjected to critical point drying in CO_2_ (Autosandri-815, Tousimis), coated with 10 nm of pure gold in a vacuum sputter coater (Desk V, Denton Vacuum) and studied using a scanning electron microscope (Jeol, JEM6610 LV) in direct mode.

### Statistical analysis

All data are expressed as the mean ± standard error mean. The flow cytometry data for ROS production are expressed as the median coefficient of two repeated and statistically reproducible measurements of at least 5 independent animals (the Friedman test). The statistical analysis was performed by one-way analysis of variance (ANOVA) using Prism software (Prism 6, GraphPad Software, Inc., San Diego, CA, USA). When the ANOVA showed significant differences, Tukey’s test was used for *post hoc* analysis. Categorical variables are presented as frequencies and were compared using the chi-square test or Fisher's exact test when appropriate. The differences were considered significant when p < 0.05.

## Results

### Identification and quantification of phenolic acids by LC/MS

LC/MS analyses provided the concentrations of ferulic acid (22.6 μg/mg), gallic acid (142.1 μg/mg) and quercetin (7.3 μg/mg) in RV.

### Determination of the antioxidant activity of *Virola oleifera*

The RV showed a high antioxidant activity, as determined by the scavenging of DPPH radicals [20], (AAI: 8.96 ± 0.14) compared with the standards ferulic acid (IC_50_: 7.12 ± 0.39 μg/ml e AAI: 5.49 ± 0.30) and BHT (IC_50_ = 8.6 ± 0.38 μg/ml e AAI 4.47 ± 0.22).

### The effect of resin from *Virola oleifera* on the attenuation of renal dysfunction after CIN in mice

Table 1 summarizes the number of deaths per group and the mean values of the biochemical indices of renal function. The experimental mouse model of CIN had a death rate of 40% (p<0.05, when compared to control), whereas the conventional treatment with NAC showed only 25% morbidity. Interestingly, the RV reduced the number of deaths per group in a dose-dependent manner; approximately 90% of the animals treated with the highest dose survived. The CIN group exhibited significantly higher (~4-fold) plasma concentrations of urea and creatinine compared with control animals (66 ± 2 and 0.16 ± 0.03 mg/dL, respectively, p<0.05). The RV also showed a dose-dependent decrease in azotemia, reducing it significantly only at the highest dose (V300: 136 ± 22 and 0.12 ± 0.03 mg/dL, p<0.05). The highest dose of RV was more effective than the conventional treatment with NAC (250 ± 50 and 0.44 ± 0.15 mg/dL). We observed a decrease in the number of viable cortex and medullary cells in the CIN group compared with control animals (CIN: 96.6±0.3 and 96.2±0.3, respectively, vs. Control: 98.0±0.1 and 98.5±0.2%, respectively, p<0.05). Interestingly, only the highest dose of RV (V300) was able to restore the cell viability to normal (cortex: 98.3±0.3 and medulla: 98.5±0.3%, p<0.05). Once again, the highest dose of RV was more effective than the standard treatment with NAC.

**Table 1:** Number of deaths per group, biochemical serum and cell viability parameters in the six experimental groups.

Parameters	Control (n = 9)	CIN (n = 9)	NAC (n = 6)	V10 (n = 9)	V100 (n = 9)	V300 (n = 7)	P
Body weight (g)	37±2	40±1	38±1	37±1	38±1	40±2	0.65
Number of deaths/group	0%	40%*	25%	44%*	23%	12%	0.01
Blood urea (mg/dL)	66±2	259±31*	250±50*	244±33*	209±34*	136±22^#^	<0.0001
Serum creatinine (mg/dL)	0.16±0.03	0.63±0.17*	0.44±0.15	0.41±0.13	0.32±0.06	0.12±0.03^#^	<0.005
Viability of cortex renal cells (%)	98.0±0.1	96.6±0.3*	97.2±0.3	96.7±0.5	97.9±0.2	98.3±0.3^#^	0.0002
Viability of medullary renal cells (%)	98.5±0.2	96.2±0.3*	96.9±0.4	95.3±0.6*	97.6±0.3	98.5±0.3^#^	<0.0001

The values are presented as the mean ± SEM for n = 6 to 9 animals per group.

* p < 0.05 vs. Control group.

^#^ p < 0.05 vs. CIN group.

### ROS production

**Fig 1:**
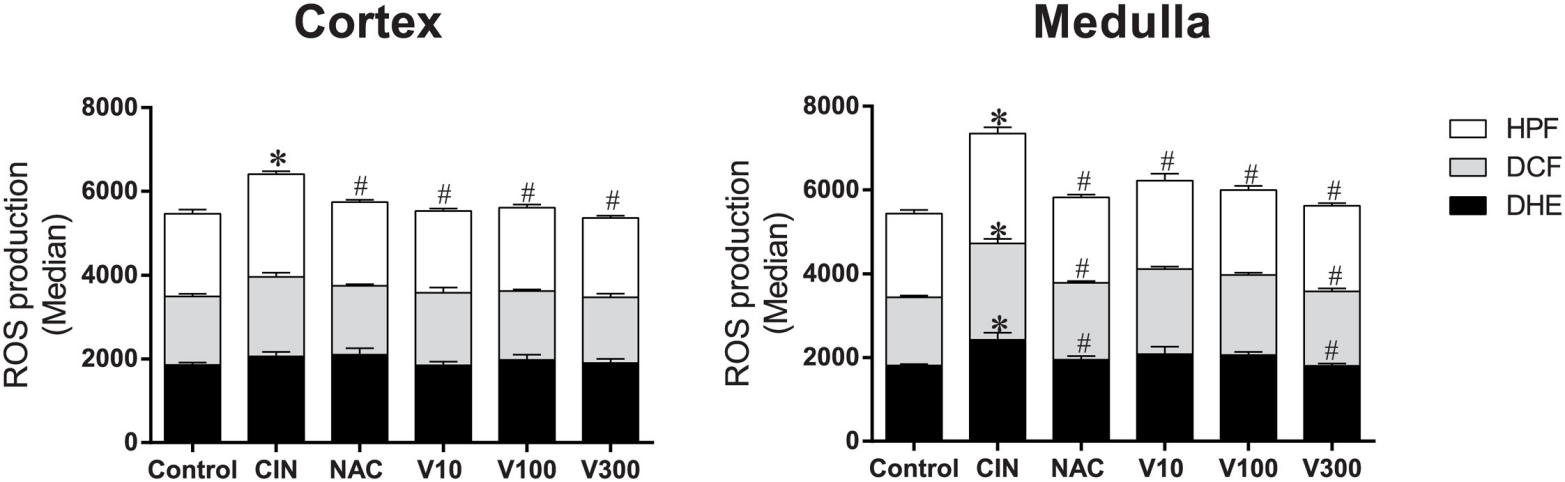
Reactive oxygen species (ROS) production. Results from cytometry analysis with dihydroethidium (DHE), 2',7'-dichlorofluorescein (DCF) and 2-[6-(4'-hydroxy)phenoxy-3H-xanthen-3-on-9-yl]benzoic acid (HPF). Bar graphs showing that after CIN, renal medulla cells (n = 6) are more sensitive to oxidative stress than cortex cells (CIN group (n = 6) compared with the control group (n = 5)). The V10 (n = 3) and V100 (n = 5) groups show a slight antioxidant effect in the two cell types, whereas NAC (n = 6) and V300 (n = 6) mainly show antioxidant effects in the medulla. The values are presented as the mean ± SEM. *p <0.05 vs. the control group and #p <0.05 vs. the CIN group.

As summarized in the bar graphs in Fig 1, we used flow cytometry with DHE, DCF and HPF to quantify the production of •O_2_^-^, H_2_O_2_ and •ONOO^-^/•OH^-^, respectively, which are reported as median fluorescence intensity (MFI, in a.u.). As expected, we observed a marked increase in ROS production in the CIN group, especially in the medulla (DHE: 34%, DCF: 40%, HPF: 32%), compared with control mice (DHE: 1808 ± 34, DCF: 1633 ± 45, HPF: 1996 ± 86 a.u., p<0.05). In the cortex, there was a significant difference only in •ONOO^-^/•OH^-^ production (+25%) compared with control mice (1965 ± 102 a.u., p<0.05). Based on previous experiments, which showed that protecting renal tubular cells against oxidative stress is an important way to avoid radiocontrast nephropathy [3,9], we evaluated intracellular ROS levels in renal cortex and medulla tissues in all groups of treated animals. Interestingly, the highest dose of RV (V300) restored the three types of ROS to basal levels in the medulla (DHE: 1802 ± 52, DCF: 1782 ± 75, HPF: 2041 ± 57 a.u., p<0.05) and was as effective as the conventional treatment with NAC (DHE: 1947 ± 86, DCF: 1840 ± 45, HPF: 2035 ± 65 a.u., p<0.05). In renal cortex tissue, we observed a small reduction in oxidative stress only for •ONOO^-^/•OH^-^ production for both the conventional treatment with NAC and the different doses of RV.

### Apoptosis

**Fig 2:**
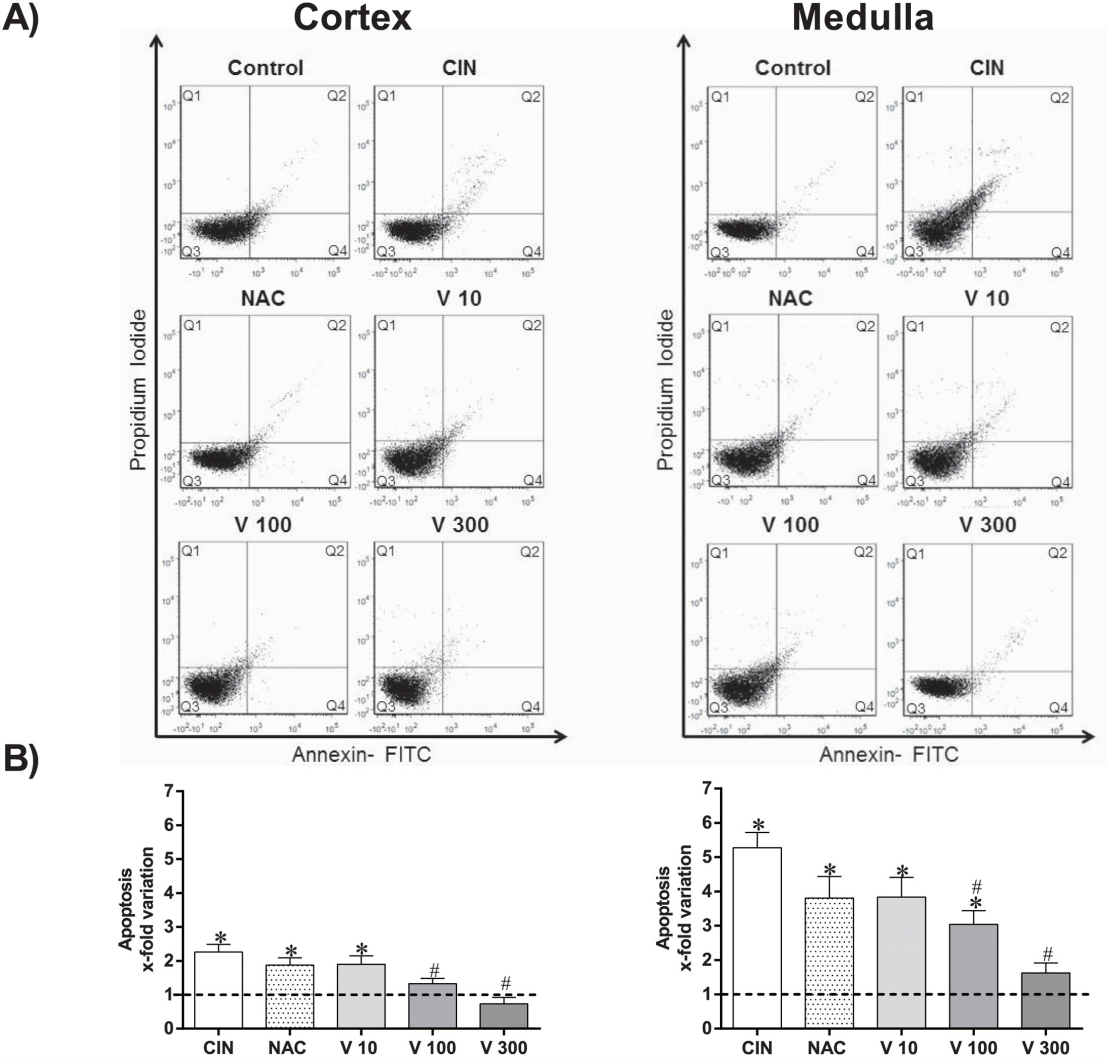
Flow cytometry analysis of apoptosis. A) Dot plots showing the apoptosis ratios of kidney cells from Control (n = 4), CIN (n = 6), NAC (n = 6), V10 (n = 4), V100 (n = 4) and V300 (n = 4) groups subdivided into cortex (left panel) and medulla cells (right panel). The apoptosis ratios were determined using propidium iodide (PI) and FITC-annexin V. The Q2 + Q4 quadrants represent the cells that are in apoptosis. Note the remarkable decrease in the number of apoptotic cells (Q2 + Q4) in the V300 group. (B) The bar graph shows the apoptotic indices (Q2 + Q4) expressed in x-fold variation relative to the Control group. The values are presented as the mean ± SEM. *p <0.05 vs. the Control group and #p <0.05 vs. the CIN group.

Apoptosis was evaluated in the same tissues using PI and annexin V staining and flow cytometry analysis. Fig 2 shows representative dot plots for each group. A clear increase in the number of apoptotic cells (Q2 + Q4) was observed in the CIN group (medulla: ~5-fold; cortex: ~2-fold) compared with control mice (medulla: 0.6 ± 0.1; cortex: 1.2 ± 0.1%, p<0.05). In addition to the antioxidant effects, the doses of RV showed dose-dependent antiapoptotic effects in both the renal medulla (V10: ~4-fold, V100: ~3-fold and V300: ~2-fold) and cortex (V10: ~2-fold; V100: ~1.5-fold and V300: ~1-fold). The V100 and V300 groups showed a significant reduction in apoptosis compared with the CIN group (p<0.05). Finally, when compared with the conventional treatment, NAC, the V300 dose was more effective in reducing apoptosis (medulla: ~4-fold and cortex: ~2-fold, p<0.05).

### Kidney morphometric parameters

**Fig 3:**
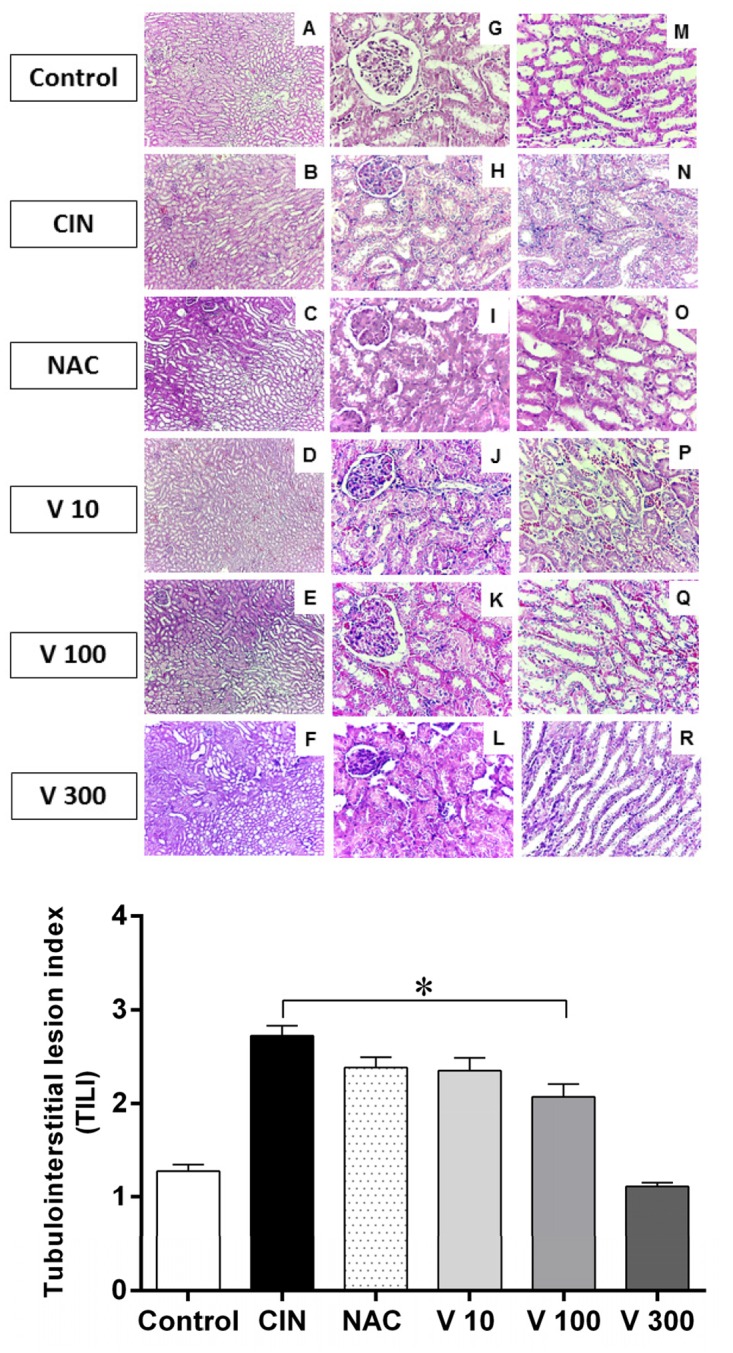
TILI index. Resin from *Virola oleifera* ameliorates tubulointerstitial lesions in radiocontrast-induced nephropathy in mice. Top panel: Representative micrographs showing kidney histology in different groups of mice 24 h after induction of CIN. The kidney sections were stained with hematoxylin and eosin (HE) reagents. A-F: the cortex-medulla section (100x); G-L: the cortical region (400x) and M-R: the medulla region (400 x) showing increased epithelial cell shedding, tubular dilation and tubulointerstitial lesions. Bottom panel: The tubulointerstitial lesion (TILI) indices of the different groups: Control (n = 4), CIN (n = 4), NAC (n = 5), V10 (n = 7), V100 (n = 8), V300 (n = 4). The values are presented as the mean ± SEM. *p <0.05 vs. Control group.

As summarized in Fig 3, histological assessment of the kidneys showed that the CIN group exhibited remarkable alterations in the typical renal tubular architecture (Fig 3, top panel). The CIN group showed increased epithelial cell shedding, tubular dilation and more tubulointerstitial lesions (2-fold) when compared with control mice (CIN: 2.7 ± 0.11 vs. Control: 1.3 ± 0.07, p<0.05) (Fig 3, bottom panel). Interestingly, the RV significantly reduced these lesions only at the highest dose (V300: 1.1 ± 0.04, p<0.05). Treatment with NAC (2.4 ± 0.11) or with lower doses of RV did not protect against acute tubular injury (V10: 2.4 ± 0.13; V100: 2.0 ± 0.14).

**Fig 4:**
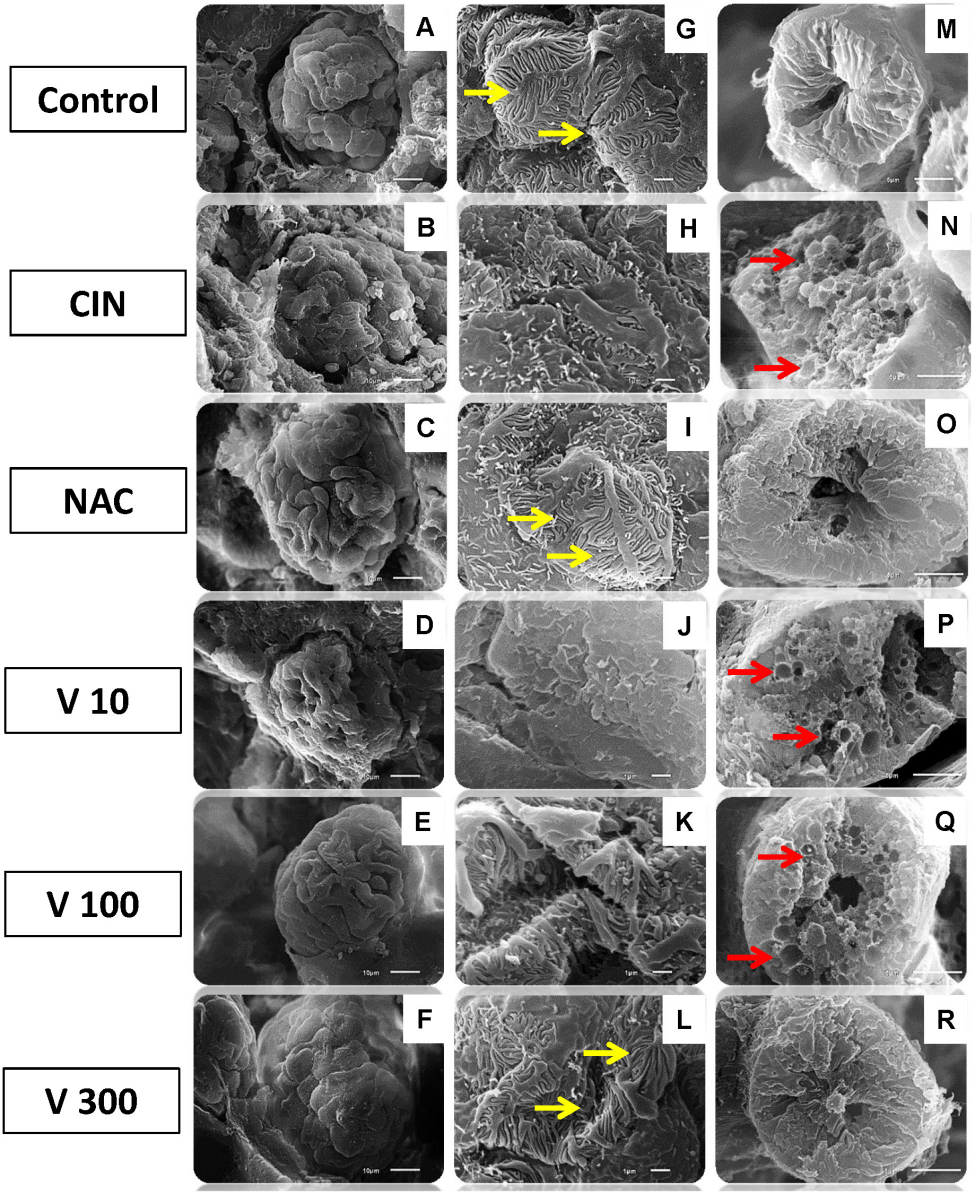
Scanning electron microscopy (SEM) photographs. Freeze-fractured kidney tissue samples from different groups confirming the decrease in renal glomerular and tubular injuries. The first column (A-F, scale bar = 10 μm) shows scanning images of whole glomeruli, showing structural preservation of the surface tissues in the NAC (C) and V 300 (F) groups, similar to the Control group. The CIN (B), V10 (D) and V100 (E) groups exhibited shrunken glomerular tufts accompanied by loss of structural cohesion. The second column (G-L, scale bar = 1 μm) shows higher magnification of podocytes to display the primary processes and the interdigitating secondary processes. The CIN (B), V10 (D) and V100 (E) groups showed atypical podocytes. Only the NAC (I) and V300 (L) groups were similar to the control structures (G), with smooth foot processes that tightly apposed each other (yellow arrows). The third column (M-R, scale bar = 5 μm) shows scanning images of proximal tubules with normal structure (M), with vacuolization and luminal congestion (red arrows) after radiocontrast nephropathy (N, P and Q) and attenuation of cytoplasmic vacuoles in the NAC (O) and V300 (R) groups.

Fig 4 shows typical scanning electron microscopy (SEM) photomicrographs confirming the results obtained in our previous experiments. In the CIN group, we observed a shrunken glomerular tuft, loss of structural cohesion, atypical podocytes, luminal congestion and vacuolar degeneration of tubular epithelial cells (Fig 4, images B, H and N). Interestingly, only the NAC (Fig 4, images C, I and O) and V300 (Fig 4, images F, L and R) groups showed attenuation of the iodinated contrast-induced lesions in both glomerular podocytes and tubular cells.

## Discussion

The present study is the first to investigate the effects of RV on both renal function and structure after oxidative stress in an innovative radiocontrast-induced nephrotoxicity model in mice. We found using biochemical markers and flow cytometry that the highest dose of RV (V300) protects against the development of renal dysfunction and decreases injury due to oxidative stress after CIN. Moreover, RV markedly decreased the incidences of renal glomerular and tubular injuries, as shown by histological analysis.

The development of novel candidate renoprotective substances for the prevention of CIN has been hindered by the lack of a reproducible and simple experimental model. It is known that murine animals, and likewise healthy humans, are resistant to contrast-induced nephropathy [16]. In our mouse model, we used the contrast medium at a dosage that is comparable to clinical use (1.5 g/kg iodine in routine angiographic practice). In addition, we used prostaglandin and NO inhibitors to mimic the clinical conditions that usually predispose a patient to CIN, increase medullary hypoxic stress and ultimately lead to a decline in kidney function [3,11,21,29]. Interestingly, we observed an increase in serum urea and creatinine concentrations in the CIN group, confirming the unprecedented success of CIN in the Swiss mouse model but not in CIN mice supplemented with the highest dose of RV. This finding suggests that RV treatment can prevent the renal dysfunction caused by iodinated contrast media in a dose-dependent manner and consequently improve survival rates. Previous studies have shown that conventional strategies for pharmacological prophylaxis of CIN include vasodilators and/or antioxidant agents, but several clinical trials have yielded conflicting results [9,11,12,30,31]. Alternatively, NAC (a glutathione precursor) has been used to prevent CIN because of its antioxidant properties, low risk of adverse events and low cost [9,11,12,30,31], which justifies the inclusion of this group as a standard treatment. It should be noted that the decrease of azotemia in the V300 group was more pronounced than that in the NAC group, demonstrating an important potential role for RV in protection against contrast-induced renal injury. Considering that oxidative stress is one of the key mechanisms underlying pathogenesis in CIN [2] and that RV displays high antioxidant properties [19], we decided to investigate oxidative stress-related parameters in the kidneys of all the groups.

Recent studies have reported several indirect markers of oxidative stress in CIN, such as increased lipid peroxidation [7,11,28], impairment of the antioxidant defense system [11,32] and/or increased synthesis of prooxidant enzymes [7,16]. The present study is the first to report ROS overproduction (~20%) in kidneys in a CIN experimental model using fluorescent probes as direct measurements [33]. Although the magnitude of differences between our non-treated and treated groups was small, this innovative method has the advantage of identifying the participation of non-cumulative ROS. In contrast, studies using the traditional indirect methods have detected higher differences, probably because they assay the cumulative oxidation of target molecules (e.g., lipids, proteins or DNA) [10,23,25,28,52] without distinguishing among the direct contributors to oxidative stress. Therefore, our results contain two important findings. First, our data describes the distinct participation of •O_2_^-^, H_2_O_2_ and mainly •OH/OONO^-^ species, thereby emphasizing the important contribution of oxidative stress in the CIN experimental mouse model. We speculate that the preponderance of the peroxynitrite species may occur due to its high stability, which allows it to participate directly in cytotoxicity during injury [34–36]. Second, our data revealed that oxidative stress occurs in the whole kidney but most intensely in the medulla, the original site of CIN due to hypoperfusion and consequent mitochondrial damage [8,37]. Third, another novel finding of the present study is that the RV (V300) abolished the difference in ROS production observed between the Control and CIN groups. The highest dose of RV performed as well as the antioxidative effect of the standard NAC. Thus, the anti-ROS effect observed for V300 corroborates our *In Vitro* antioxidant analysis (DPPH•) and other recent studies that have linked nephroprotection to the presence of phenolic [38,39] and flavonoid compounds [22,40], which are abundant in the RV [19].

Previous data from our laboratory [22,23,41] and from others [6,42–44] have shown that ROS regulate the pathways linked to apoptosis and contribute to glomerular and tubular injury. At the same time, other recent studies have demonstrated that contrast medium has direct toxic effects on renal tubular cells, such as depolarization of the mitochondrial membrane (contributing to ROS production), DNA fragmentation, apoptosis and cellular morphologic changes [3,4,45,46]. We hypothesized that antioxidant RV could inhibit renal cell apoptosis after CIN, and therefore protect against the kidney dysfunction induced by contrast medium. As expected, our flow cytometry data showed that this CIN model is accompanied by apoptosis in the whole kidney, although the medulla is the primary target, which corroborates previous results obtained with microscopic images, apoptosis-related markers or cytotoxicity assays [4,10,16]. Most importantly, here we demonstrate for the first time that RV was able to greatly decrease apoptosis and restore cell viability in a dose-dependent manner, with greater protective effects than those of the NAC group. Thus, our data identify two important characteristics of the resin from *V*. *oleifera*: in addition to its renoprotective effects, it shows no evidence of cytotoxicity even at the highest dose (V300) and supported by our recent observation of a high LD_50_ (2500 mg/kg) (data not shown), which demonstrates its potential for use in future clinical investigations.

It is known that after filtration, iodinated contrast media are concentrated inside the renal tubules, directly exposing the tubular cells, which worsens the medium’s damaging effects [2]. In addition to the biochemical and flow cytometry parameters we studied, our histopathological HE and SEM data agreed with other reports [4,10,16,32,47] and showed a marked disruption of renal structure in the CIN group compared with control animals. Interestingly, in addition to its direct antiazotemic, antioxidative and antiapoptotic properties, we demonstrated that RV also exhibits a nephroprotective effect by ameliorating the pathological changes in glomeruli structure and vacuolization in a dose-dependent manner. In agreement with other recent reviews of herbal studies [48,49], we speculate that these renoprotective effects of RV may be exerted, at least in part, by a pool of antioxidants contained in this species, as indicated by the presence of ferulic acid [50], gallic acid [51] and quercetin [22,52], justifying its superiority compared with NAC, a conventional antioxidant used in clinical treatment. However, considering the other intrinsic properties of RV [19,53] and its analogous species [54], we cannot rule out other pathways involved directly or indirectly in the restoration of mitochondrial number and function [55], anti-inflammation, immunomodulation, improvement of tubular regeneration capacities through proliferation and migration or prevention of the differentiation process [52,56].

A limitation of our study is that we did not analyze other renal parameters such as proteinuria or cystatin C, which are potential biomarkers for tubular damage and impaired renal function. In future studies, we intend to investigate the effects of RV on physiological parameters, such as renal blood flow and glomerular filtration rate, in this novel experimental Swiss mouse model of CIN.

In summary, the present study demonstrates, for the first time, the renal protective role of resin from *Virola oleifera*, which reduces the renal dysfunction and morphological tubular injury caused by CIN induction in mice. These renoprotective effects are accompanied by antioxidant and antiapoptotic activity. Thus, this resin may be a promising therapeutic agent that could be used for the prevention and/or treatment of renal dysfunction induced by contrast media. Further clinical trials are needed to establish efficacy, tolerability and pharmacokinetics of this natural extract.
